# Risk Factors Associated with Cardiac Autonomic Modulation in Obese Individuals

**DOI:** 10.1155/2020/7185249

**Published:** 2020-03-26

**Authors:** Camila Oliveira, Erika Aparecida Silveira, Lorena Rosa, Annelisa Santos, Ana Paula Rodrigues, Carolina Mendonça, Lucas Silva, Paulo Gentil, Ana Cristina Rebelo

**Affiliations:** ^1^Medicine Faculty, Health Science Post-Graduation Program, Universidade Federal de Goiás, Goiânia, Goiás, Brazil; ^2^Medicine Faculty, Coordinator of the Severe Obesity Study Group, Federal University of Goiás, Goiânia, Brazil; ^3^College of Physical Education and Dance, Federal University of Goiás, Goiânia, Brazil; ^4^Department of Morphology, Biological Sciences Institute, Federal University of Goiás, Goiânia, Goiás, Brazil

## Abstract

Obesity leads to an imbalance in the autonomic nervous system, especially in increased sympathetic modulation and decreased vagal tone, and some anthropometric, metabolic, and lifestyle variables may increase the risk of developing cardiovascular disease. *Objective*. To analyze the association between cardiovascular autonomic modulation and biochemical and anthropometric markers, food intake, and physical activity level in severely obese individuals. *Methodology*. The present study is a cutout of a randomized clinical trial “Effect of nutritional intervention and olive oil in severe obesity” (DieTBra Trial), where the baseline data were analyzed. Anthropometric data, biochemical exams, heart rate variability (HRV), accelerometry, and 24 h recall (R24H) of obese patients (body mass index BMI ≥35 kg/m^2^) were collected. *Results*. 64 obese patients were analyzed, with a mean age of 39.10 ± 7.74 years (27 to 58 years). By HRV analysis, in the frequency domain, the obese had a higher predominance of sympathetic autonomic modulation (low frequency (LF) 56.44 ± 20.31 nu) and lower parasympathetic modulation (high frequency (HF) 42.52 ± 19.18 nu). A negative association was observed between the variables Homeostasis Evaluation Model (HOMA-IR) and HF (*p* = 0.049). In the physical activity analysis, there was a negative association between moderate to vigorous physical activity and the sympathetic component (*p* = 0.043), and for sedentary time (ST), there was a negative association with HF (*p* = 0.049) and LF/HF (*p* = 0.036) and a positive association with LF (*p* = 0.014). For multiple linear regression, waist circumference (WC) and HOMA-IR values were negatively associated with HF (*β* = −0.685, *p* = 0.010; *β* = −14.989, *p* = 0.010; respectively). HOMA-IR (*β* = 0.141, *p* = 0.003) and the percentage of lipids ingested (*β* = −0.030, *p* = 0.043) were negatively associated with LF/HF. *Conclusion*. Among the cardiovascular risk variables studied, insulin resistance and central adiposity showed the greatest influence on cardiac autonomic modulation of obese, increasing the risk for cardiovascular disease.

## 1. Introduction

Studies show that obesity leads to the development of noncommunicable diseases such as hypertension, hypercholesterolemia, insulin resistance, and type 2 diabetes [1–3], and also several previous studies have found changes in cardiac autonomic modulation in overweight and obese individuals [4–8], which may be more affected in obese individuals (body mass index (BMI) ≥35.0 kg/m^2^) [9]. This impairment occurs through an imbalance in the autonomic nervous system (ANS), especially in increased sympathetic modulation and decreased vagal tone leading to decreased heart rate variability (HRV) [5, 7, 10].

The measurement of these patterns can provide a sensitive and early indicator of serious health impairment in obese individuals with a higher risk of multimorbidity and early mortality [9], as they can be indicators of risk of death and cardiovascular disease [2, 11, 12], such as coronary artery disease, myocardial infarction, sudden death, heart failure and arrhythmias [7, 13].

In the literature, there are some studies that show the direct association between sympathetic and/or parasympathetic components and risk factors in obese individuals. Kiviniemi et al. showed an association between reduced parasympathetic modulation and glucose [14]. Vagal modulation was also inversely associated with body fat percentage [15] and with high body mass [16] and waist circumference (WC) [17, 18].

Other studies indicate the sympathetic predominance of heart rate in obese, in which there was a negative correlation with Homeostasis Evaluation Model (HOMA-IR) [19], where BMI and peripheral obesity measured by forearm anterior fold demonstrated a positive correlation with low frequency (LF) [20]. Overweight individuals had sympathovagal imbalance due to increased sympathetic activity associated with visceral fat [4]. Physical activity significantly reduced adiposity rates and improved HRV variables related to vagal modulation in sedentary obese individuals [21].

Risk factors are frequently researched, as in the study by Santos et al., cardiometabolic risk factors such as dyslipidemia, HOMA-IR, and hypertension were found in the severely obese patients surveyed, with a significantly higher prevalence in patients with a BMI ≥45 kg/m^2^ [22]. However, no study has comprehensively evaluated which HRV indices are associated with cardiovascular risk variables in severely obese subjects, remaining inconclusive among anthropometric (BMI and WC), metabolic (dyslipidemia, hypertension, HOMA-IR, glucose, and insulin), and lifestyle variables (PA, sedentary time (ST), nutritional factors, and eating habits) associated with cardiac autonomic dysfunction.

Therefore, it is essential to determine risk factors for determining cardiac autonomic dysfunction in severely obese individuals, in order to help develop strategies for preventing cardiovascular disease in severely obese individuals. Given this scenario, the objective of the present study was to analyze the association between cardiovascular autonomic modulation and anthropometric, metabolic, and lifestyle variables in severely obese individuals. And the hypothesis of the present study is that the increase in fat concentration may alter the changes in cardiac autonomic modulation and may be associated with other factors of obesity comorbidities.

## 2. Materials and Methods

### 2.1. Study Design and Ethical Aspects

The present study is a clipping from a randomized clinical trial “Effect of nutritional intervention and olive oil in severe obesity” (DieTBra Trial) (recorded at ClinicalTrials.gov: NCT02463435), where baseline data were analyzed.

Data collection took place at the Clinical Research Unit, Hospital das Clínicas, Federal University of Goiás (UPC/HC/UFG), Goiânia, Goiás, Brazil, from June 2015 to February 2016. This research was developed according to the recommendations of Resolution No. 466 of December 12, 2012, of the National Health Council (Brazil, 2012) and approved by the UFG HC Research Ethics Committee (CEP/HC/UFG), under protocol 747.792/2014. All patients who agreed to participate signed the informed consent form.

### 2.2. Sample Calculation

The sample calculation was estimated from GPower software, version 3.1, based on the mean and standard deviation of HRV indices (HF and LF/HF) obtained in our pilot study. For an alpha of 0.05 and 95% power, the recommendation was 62 volunteers.

### 2.3. Target Population and Selection Criteria

Patients with obesity above grade II BMI ≥35 kg/m^2^ were selected and referred to the Nutrition and Serious Obesity Outpatient Clinic (ANOG/HC/UFG) by the Unified Health System (SUS). Inclusion criteria were patients of both sexes, aged between 18 and 65 years, and BMI ≥35 kg/m^2^, calculated based on the following formula: body mass ÷ [height^2^] (kg/m^2^). We excluded patients after bariatric surgery, patients with weight loss >8% in the last trimester, who had received nutritional treatment in the last two years, were using weight loss or anti-inflammatory drugs, patients with HIV/AIDS, heart/kidney/liver failure, obstructive chronic disease, lung disease, and cancer, pregnant women, nursing mothers, and people with physical or mental disabilities. The female volunteers were assessed between the 7th and 10th days after the start of menstruation, and those who reported using oral contraceptives (with active pills followed by placebo pills) for more than 12 months were assessed during the placebo phase of the medications.

### 2.4. Data Collection

All volunteers were evaluated at the same time of day to avoid different responses of physiological variables due to circadian changes. Anthropometric data, biochemical examinations, HRV, accelerometry, and 24 h recall (R24H) were collected.

### 2.5. Anthropometry

To measure the current body mass, a Welmy digital platform scale with a capacity of 200 kg and 100 g accuracy was used. Height was performed on a stadiometer already attached to the digital scale, with an accuracy of 0.1 cm. Body mass and height data were used to calculate BMI, later classified according to WHO (2000) [23].

### 2.6. Biochemical Tests

Biochemical exams were collected after 12 hours of fasting, in the morning, before the other collection protocols were performed at UPC/UC/UFG and analyzed at Rômulo Rocha Laboratory (HC/UFG). Total cholesterol (TC), high-density lipoprotein (HDL), low-density lipoprotein (LDL), very-low-density lipoprotein (VLDL), triglycerides (TG), and Homeostasis Evaluation Model (HOMAR-IR) were analyzed.

### 2.7. Physical Activity Assessment

The PA level was assessed using the Actigraph model wGT3X accelerometer. The equipment was used for 6 consecutive days, 24 hours a day, being affixed to the nondominant wrist. Patients were instructed not to remove the device for sleeping, bathing, or swimming, as the equipment is light, small, and waterproof. WGT3X records acceleration on three axes (*x*, *y*, and *z*) at an amplitude of ±8 g and a frequency of 30 Hz. The data collected were downloaded using ActiLife 6.11.7 software and patients with available data of at least 50% of the time of use were included in the analysis. The measures used were moderate to vigorous physical activity (MVPA) (>100 mg) lasting at least 10 consecutive minutes per week and sedentary time (ST) (<50 mg) in minutes per day. In the PA level classification, the WHO (2010) recommendation was considered [24] where adults need to perform ≥150 minutes/week of MVPA.

### 2.8. 24-Hour Recall

In this research, the food consumption was evaluated by the average of three R24H in an interval of seven days, being two presential and one via telephone contact. The recall was applied by nutritionists and trainees and consisted of the definition and quantification of all foods consumed the day before. The foods consumed were listed and then asked in detail the size and volume of portions consumed, home measures used, mode of preparation, oil addition, and use of sugars or sweetener [25].

### 2.9. Heart Rate Variability

For the HRV collection, the volunteers were instructed to remain in the supine resting condition and sitting for an approximate period of 10 minutes, preventing them from moving. Heart rate (HR) was continuously monitored by a heart rate monitor through a chest strap (v800 Polar, Finland). RR interval recordings (iRR) were analyzed from Kubios HRV Analysis software, software version 2.2.

The experiments were carried out in an air-conditioned room, where the ambient temperature was artificially controlled, from an air conditioner, with a temperature between 22°C and 24ºC and relative humidity between 50 and 60%. Minimum traffic of people in the environment during the execution of the experiment was maintained.

Each subject was advised not to drink alcohol and/or stimulants 24 hours before and on the day of the tests; not to perform moderate or intense PA on the day before its application; and avoid copious meals and eat a light meal at least two hours before testing.

The iRR variations were used for the linear method analysis in the frequency domain indices, whereby the main HRV evaluation method is made by the spectral analysis that evaluates the autonomic function. The components analyzed were HF (high frequency), ranging from 0.15 to 0.4 Hz, and LF (low frequency), ranging from 0.04 to 0.15 Hz. LF is considered an index related to sympathetic and vagal modulation in the sinus node, whereas HF is the index related to vagus nerve activity over the heart, and the LF/HF ratio is used as an indirect index of sympathovagal balance [26, 27]. Measurements of these components were expressed in normalized units (nu).

### 2.10. Statistical Methods

First, a descriptive analysis was performed: absolute and relative frequency, mean, and standard deviation. The Kolmogorov–Smirnov test was applied to verify the normality of continuous variables and the Levene test was used to verify the homogeneity of the variables. All variables were considered normal and/or homogeneous. Simple linear regression was performed to identify the association between cardiovascular autonomic modulation variables (HF (nu), LF (nu), and LF/HF) and BMI (kg/m^2^), WC (cm), HOMA-IR (mg/dl), insulin (um), blood glucose (mg/dl), MVPA (min/week), ST (min/day), total energy value (TEV) (kcal/day), macronutrients (%), and SBP and DBP (mmHg). Variables with *p* < 0.20 in the simple linear regression were entered in the multiple linear regression (MLR). MLR was performed between the frequency domain indices and the adjusted variables WC (cm), HOMA-IR (mg/dL), insulin (ui), blood glucose (mg/dL), MVPA (min/week), TS (min/day), VET (kcal/day), carbohydrates (%), and lipids (%). Statistical analysis was performed in the program Statistical Package for the SocialSciences (SPSS; IBM Corp., NY, USA), version 20.0. Statistical significance was previously established at *p* < 0.05.

## 3. Results

**Figure 1:**
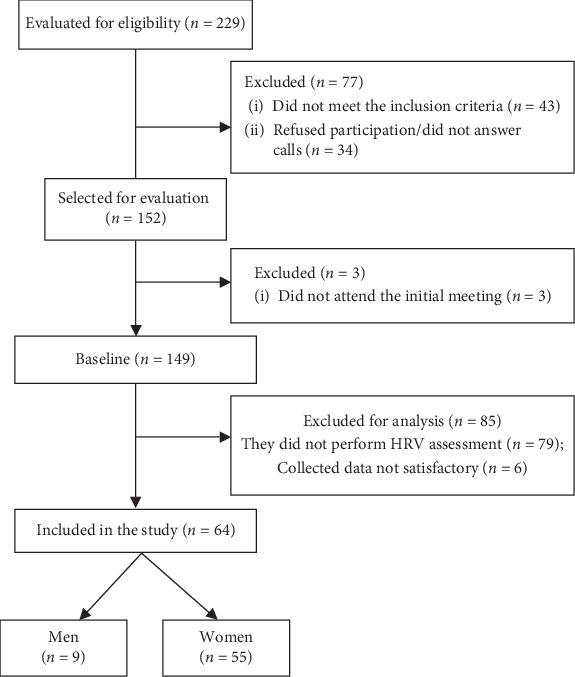
Diagram of the flow of selection and continuity of study participants.

Of the 64 obese patients analyzed in the present study, 9 were male (14.06%) and 55 female (85.93%), as shown in Figure 1, with a mean age of 39.10 ± 7.74 years (27 to 58 years). Regarding food intake, for the TEV, an average of 1956.76 ± 1097.21 kcal/day was obtained and the amount of macronutrients consumed as a percentage for carbohydrates (53.39 ± 10.78), proteins (17.90 ± 6.09), and lipids (28.1 ± 8.70).

The biochemical results of blood are shown in Table 1. For the anthropometric data evaluated, a mean BMI of 46.61 ± 6.86 kg/m^2^ was observed, with a more frequent obesity degree of 40 to 49.9 kg/m^2^ (60.93%). The mean WC was 118.83 ± 10.66, with the highest risk for all men and 84.37% of women. Patients were shown to be insulin-resistant (HOMA-IR 6.03 ± 4.10 mg/dl). The amount of minutes spent per day on ST and MVPA in minutes per week is also shown in Table 1.

**Figure 2:**
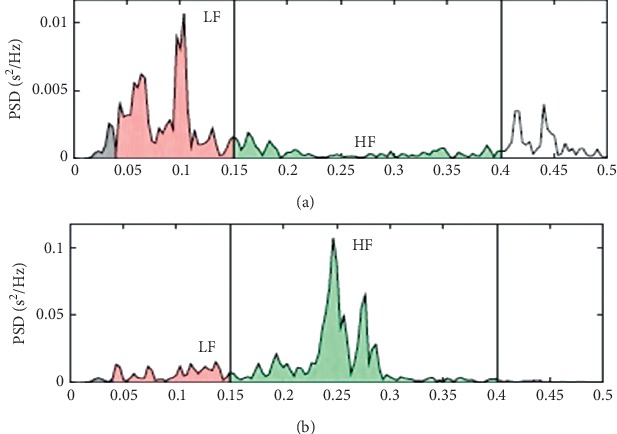
Graph in normalized unit with LF and HF values comparing (a) a severely obese woman: 34 years, weight 94 kg, height 1.62 m, and body mass index 35.8 kg/m^2^, and (b) and a nonobese woman: 35 years, weight 59.4 kg, height 1.59 m, and body mass index 23.6 kg/m^2^; HF = high frequency; LF = low frequency.

The HRV analysis results from the frequency domain linear analysis showed that the severely obese had a higher predominance of sympathetic autonomic modulation (LF 56.44 ± 20.31 nu) and lower parasympathetic modulation (HF 42.52 ± 19.18 nu) in Figure2)we can see an example of comparison between the data of two individuals, one obese and the other non-obese, from the analysis in the frequency domain.

From the simple linear regression analysis, the BMI, WC, TEV, carbohydrate (%), lipid (%), protein (%),SBP, and DBP were not associated with cardiovascular autonomic modulation (*p* > 0.05) (Table 2). However, a negative association was observed between the variables HOMA-IR and HF (*p*=0.049), and HOMA-IR and LF/HF (*p* ≤ 0.001). For insulin and glycemia, there was a negative association with sympathovagal balance (*p*=0.002 and *p*=0.021, respectively). In the analysis of PA, there was a negative association between MVPA and the sympathetic component (*p*=0.042), and for the ST, there was a negative association with HF (*p*=0.049) and LF/HF (*p*=0.036) and a positive association with LF (*p*=0.014).

In Table 3, from the multiple linear regression, the WC and HOMA-IR values were negatively and significantly associated with HF (*p*=0.010). HOMA-IR and percentage lipid values were negatively associated with LF/HF (*p*=0.003 and *p*=0.043, respectively). No associations were observed between insulin, glycemia, MVPA, ST, TEV, and carbohydrate (%) with autonomic modulation.

## 4. Discussion

Among the cardiovascular risk factors analyzed in the present study, it was found that insulin resistance and central adiposity were independently associated with changes in HRV parameters in the frequency domain (LF, HF, and LF/HF) and that these altered factors in the population in question have a strong relationship with the dysregulation in cardiac autonomic function and may be the mechanism for the increased prevalence of cardiovascular diseases in obesity.

In the present study, it was observed a negative association between HOMA-IR and parasympathetic modulation and a positive association with global variability was observed. A similar association was reported in a study from our research group in which participants with metabolic syndrome had elevated blood glucose levels associated with reduced parasympathetic modulation [14]. Also in this perspective, insulin was inversely associated with HRV among patients with higher levels of obesity and lower levels of PA [28], since the severely obese in the present study showed a low amount of minutes spent on MVPA (98.92 ± 41.00 min/week).

Our sample showed high body mass and accumulation of visceral fat (BMI 46.61 ± 6.86 kg/m^2^ and WC 118.83 ± 10.66 cm), and in this sense, adiposity and especially central fat can lead to insulin resistance, which may alter the function of the autonomic nervous system. Another important point found in the present study was WC associated with worsening vagal tone, in which central obesity, rather than general fat, is related to the impairment of autonomic nervous system function [20]. Corroborating our data, the study by Yadav et al. observed an increase in waist-to-hip ratio strongly associated with reduced cardiac parasympathetic activity in obese individuals, and there may be a higher risk of cardiovascular morbidity and mortality due to cardiac autonomic changes [8].

Regarding food consumption, a negative association was observed between the percentage of lipids consumed and the global variability, implying that lipids exert a protective factor in cardiac autonomic function. The fatty acid profile found in dietary fat has different consequences on the human body [29]. It is well established in the literature that excessive saturated fatty acid consumption is associated with increased risk of cardiovascular disease and may reduce the risk of sudden death in cardiovascular disease patients [30], whereas ingestion of unsaturated fatty acids as monounsaturated and especially polyunsaturated fatty acids (PUFAs) has been associated with reduced cardiac mortality [31], with beneficial effect on blood lipid profile in patients with coronary artery disease, and may also be associated with a reduction in the frequency of ventricular tachyarrhythmias in implantable defibrillator cardioverter (ICD) recipients with ischemic cardiomyopathy [32], and in animal models, prevent fatal ischemia-induced cardiac arrhythmias [33] [30].

Also in a review study, La Rovere and Christensen identified that PUFAs can have positive health effects on cardiovascular control in humans, especially on parasympathetic function. One explanation would be that these fatty acids affect the expression of genes involved in inflammation and lipid metabolism [34]. For the present study, we assume that the protective effect that lipid ingestion promoted on the overall cardiovascular variability was perhaps due to the consumption of unsaturated fatty acids, since these exert a reduction in cardiovascular events.

Regarding PA, it was observed that higher levels of MVPA were associated with reduced LF; in this sense, higher levels of PA in severely obese induce a positive response of cardiac sympathetic modulation, and PA may be a cardiac protector, whereas increased sympathetic tone results in increases in heart rate and blood pressure [35], which may increase the risk of cardiovascular disease occurrences.

So being physically active reduces the risks for obesity-related cardiovascular disease by positively influencing autonomic nerve function [2, 16, 36] and insulin resistance [37], besides reducing the action of catecholamines (attenuating acetylcholine-oriented vasoconstriction), which reduce resting HR, and during submaximal exercise, allowing for faster HR recovery, which indicates improved vagal modulation and less risk for cardiovascular outcome [38, 39]. In this scenario, healthy lifestyle was associated with higher HRV, suggesting a positive effect on ANS and throughout life. PA was positively and independently related to HRV in obese adults, implying that regular physical activity induces structural and functional adaptations in the cardiovascular system [16, 40].

In the present study, for ST there was a correlation with all the frequency domain indices, in which it correlated negatively with parasympathetic component and the sympathovagal HRV balance and positively with cardiac sympathetic modulation. In line with this, the study by Lião et al. showed a higher cardiac frequency and a decrease in the HF index in overweight elderly people and a lower HRV in the group with low physical capacity compared to the group with high physical capacity [41], implicating that ST is related to increased risk of developing cardiovascular disease, in which the occurrence of a heart attack is twice as high in sedentary individuals when compared to those who are regularly active [42], all-cause mortality, and reduced life expectancy [43].

Among the limitations of our study, not having a control group for comparison with the population in question was an important factor. Nevertheless, the data presented indicate the importance to modify the lifestyle in severely obese by performing PA.

Control of risk factors in obesity is extremely important as they produce significant effects on the cardiovascular autonomic system and may be fundamental to the health of these patients. Anthropometric assessment and identification of risk factors alone or in combination can be useful for planning and implementing public policies aimed at reducing morbidity and mortality from cardiovascular disease [42]. Therefore, the information in this study is extremely relevant, since it investigated the cardiovascular risk factors that affect severely obese individuals and observed that the main factors that affect the alteration of autonomic function are insulin resistance, central adiposity, MPVA, and ST.

This demonstrates the importance of adopting strategies to change the lifestyle of the severely obese [44], mainly due to the practice of physical activity as a nonmedication strategy and primary prevention for the treatment of obesity [45], along with additional weight gain with the maintenance of weight loss of those who reach it [46]. Public health programs should be promoted with a multidisciplinary approach in which health professional can better advise the severely obese, physically, foodily and psychologically. Multidisciplinary public health policy programs should be promoted, where health professionals can better advise the severely obese, physically, foodily, and psychologically.

## 5. Conclusion

The study reveals that among cardiovascular risk factors, insulin resistance and central adiposity were independently associated with cardiac autonomic modulation in obese individuals, having fundamental importance in altering cardiac autonomic modulation of obesity.

## Figures and Tables

**Table 1:** Clinical characteristics of severely obese adults, Goiânia, Goiás.

Variables	*n* (%)	Mean ± SD
Age (years)		39.10 ± 7.74
18–29	7 (10.93)	
30–39	26 (40.62)	
40–49	23 (35.93)	
≥50	8 (12.5)	

BMI (kg/m^2^)		46.61 ± 6.86
35.00–39.9	9 (14.06)	
40.00–49.9	39 (60.93)	
>50.00	16 (25)	

Dyslipidemia		
Total cholesterol <190 (mg/dl)		192.72 ± 41.14
Yes	35 (54.68)	
No	29 (45.31)	
HDL-c >40 (mg/dl)		50.10 ± 12.89
Yes	56 (87.5)	
No	8 (12.5)	
Triglycerides <150 (mg/dl)		158.92 ± 67.00
Yes	30 (46.87)	
No	34 (53.12)	
LDL-c <70 (mg/dl)		110.26 ± 38.68
Yes	4 (6.25)	
No	60 (93.75)	
HOMA-IR (mg/dl)		6.03 ± 4.10
Normal	12 (18.75)	
Changed	52 (81.25)	
Blood glucose (mg/dl)		104.69 ± 35.52
≤99	41 (64.06)	
100–125	12 (18.75)	
≥126	11 (17.18)	
MVPA, ≥150 (min/week)		98.92 ± 41.00
Yes	4 (6.25)	
No	60 (93.75)	
Sedentary time (min/day)		1180.55 ± 81.51
Normal < 1000	1 (1.65)	
Changed ≥1000	63 (98.43)	
Hypertension ≤120/80 (mmHg)		
SBP		126.66 ± 17.02
DBP		84.33 ± 10.05
Yes	29 (45.31)	
No	35 (54.68)	
Diabetes mellitus 2		
Yes	18 (28.12)	
No	46 (71.87)	

Values expressed as mean ± standard deviation. BMI = body mass index; WC = waist circumference; HDL-c = high-density lipoprotein cholesterol; LDL-c = low-density lipoprotein; VLDL-c = very-low-density lipoprotein; HOMAR-IR = homeostasis evaluation model; MVPA: moderate to vigorous physical activity; SBP = systolic blood pressure; DBP = diastolic blood pressure.

**Table 2:** Linear regression among cardiovascular autonomic modulation variables with BMI, WC, HOMA-IR, insulin, blood glucose, MVPA, ST, TEV, percentage macronutrients, SBP, and DBP.

	HF (nu)			LF (nu)			LF/HF		
Variables	*β*	95% CI	*p* value	*β*	95% CI	*p* value	*β*	95% CI	*p* value
BMI (kg/m^2^)	2.756	−3.646–9.157	0.393	0.991	−7.286–9.268	0.812	−0.008	−0.064–0.049	0.792
WC (cm)	1.252	−2.877–5.382	0.547	4.735	−0.453–9.924	0.073	−0.014	−0.050–0.022	0.439
HOMA-IR (mg/dl)	−11.540	−23.042–−0.040	0.049^*∗*^	13.726	−1.133–28.584	0.070	−0.168	−0.253–−0.084	<0.001^*∗*^
Insulin (ui)	−3.270	−7.005–0.464	0.085	3.416	−1.426–8.259	0.163	−0.048	−0.078–−0.019	0.002^*∗*^
Blood glucose (mg/dl)	−0.762	−1.999–0.474	0.222	0.941	−0.651–2.532	0.242	−0.012	−0.023–−0.002	0.021^*∗*^
MVPA (min/day)	−0.844	−1.898–0.210	0.114	−1.389	−2.726–−0.051	0.042^*∗*^	0.005	−0.005–0.014	0.303
ST (min/day)	−0.528	0.002–1.053	0.049^*∗*^	0.837	0.173–1.502	0.014^*∗*^	−0.005	−0.010–−0.000	0.036^*∗*^
TEV (kcal/day)	0.021	−0.019–0.061	0.300	0.041	−0.010–0.092	0.110	−0.000	−0.001–0.000	0.146
Carbohydrate (%)	0.837	−4.914–3.241	0.683	−2.686	−7.892–2.521	0.306	0.028	−0.007–0.063	0.118
Lipid (%)	2.689	−2.322–7.700	0.288	5.404	−0.953–11.762	0.094	0.037	−0.081–0.006	0.091
Protein (%)	−2.868	−10.056–4.321	0.428	−2.619	−11.886–6.648	0.574	−0.012	−0.075–0.052	0.720
SBP (mmHg)	−41.830	−0.2344–0.186	0.822	42.47	−0.186–0.233	0.823	3.480	−2.305–2.870	0.825
DBP (mmHg)	−144.600	−0.141–0.122	0.912	158.9	−0.127–0.140	0.925	8.708	−1.540–1.769	0.890

HF = high frequency; LF = low frequency; LF/HF = low frequency/high frequency ratio; nu: normalized units; BMI = body mass index; WC = waist circumference; HOMAR-IR = Homeostasis Evaluation Model; MVPA = average of minutes per day spent in vigorous physical activity (>100 mg); ST = sedentary time; VET = total energy value (kcal/day), SBP = systolic blood pressure, DBP = diastolic blood pressure. ^*∗*^*p* < 0.05.

**Table 3:** Multiple linear regression between frequency domain indices and adjusted variables WC, HOMA-IR, insulin, blood glucose, MVPA, ST, TEV, carbohydrate, and lipid.

	HF (nu)			LF (nu)			LF/HF		
Variables	Adjusted *β*	95% CI	*p* value	Adjusted *β*	95% CI	*p* value	Adjusted *β*	95% CI	*p* value
WC (cm)	−0.685	0.169–1.201	0.010^*∗*^	2.792	−2.984–8.569	0.337	−0.002	−0.007–0.002	0.282
HOMA-IR (mg/dl)	−14.989	−26.273–−3.705	0.010^*∗*^	8.670	−6.480–23.819	0.257	0.141	−0.231–−0.051	0.003^*∗*^
Insulin (ui)	−0.038		0.924	0.237		0.589	−0.206		0.647
Blood glucose (mg/dl)	—		—	—		—	−0.205		0.518
MVPA (min/day)	−0.196		0.381	0.181		0.447	—	—	—
ST (min/day)	−0.318		0.169	0.687	−0.015–1.390	0.055	−0.459		0.085
TEV (kcal/day)	0.007		0.954	0.034	−0.017–0.858	0.183	—		—
Carbohydrate (%)	—	—	—	—	—	—	−0.008	−0.068–0.053	0.803
Lipid (%)	—		0.699	3.543	−2.832–9.917	0.270	−0.030	−0.106–0.046	0.043^*∗*^

LF = low frequency; HF = high frequency; LF/HF = low frequency/high frequency ratio, nu: normalized units; WC = waist circumference; HOMAR-IR = Homeostasis Evaluation Model; MVPA = average of minutes per day spent in vigorous physical activity (>100 mg); ST = sedentary time; TEV = total energy value (kcal/day). ^*∗*^*p* < 0.05.

## Data Availability

Data used to support the results of this study are available from the corresponding author upon request.
